# Serotonin Transporter Activity in Mouse Oocytes Is a Positive Indicator of Follicular Growth and Oocyte Maturity

**DOI:** 10.3390/ijms241411247

**Published:** 2023-07-08

**Authors:** Nina M. Alyoshina, Maria D. Tkachenko, Yulia O. Nikishina, Denis A. Nikishin

**Affiliations:** 1 N.K. Koltzov Institute of Developmental Biology, Russian Academy of Sciences, Vavilova Street, 26, 119334 Moscow, Russia; n.alyoshina@idbras.ru (N.M.A.); tkmadm@yandex.ru (M.D.T.); zubova.y@gmail.com (Y.O.N.); 2 Faculty of Biology, Lomonosov Moscow State University, Leninskie Gory, 1, Bld. 12, 119991 Moscow, Russia

**Keywords:** serotonin, SERT, SSRI, fluoxetine, ovary, apoptosis, proliferation, oocyte maturation

## Abstract

Serotonin (5-hydroxytryptamine, 5-HT) is known to be a regulator of oocyte maturation in a large number of animal species. In maturing mammalian oocytes, the accumulation of exogenous, maternal serotonin occurs due to the activity of the membrane transporter SERT. In this work, we investigated how SERT activity in oocytes correlates with indicators of follicular selection and oocyte maturity. An immunohistochemical study showed that the difference in the 5-HT intake activity in oocytes does not correlate with the marker of apoptosis in follicular cells, but positively correlates with markers of follicular growth, such as granulosa proliferation and follicle size. Functional analysis of oocytes at different stages of maturation showed that the expression and activity of SERT increases with oocyte maturation. An in vivo experiment on administration of the selective serotonin reuptake inhibitor fluoxetine (20 mg/kg) for 7 days showed a significant decrease in the content of serotonin in both growing GV-oocytes and ovulated mature MII-oocytes. The data obtained clearly indicate that the mechanism of specific membrane transport of serotonin normally ensures the accumulation of serotonin in maturing oocytes, and can be considered as a promising positive marker of their mature status.

## 1. Introduction

The maturation of mammalian oocytes is a complex process that involves inner and outer molecular signaling. The list of factors associated with oocyte competence and maturity continues to grow. Some of the most well-established markers are related to the morphology of oocyte (homogeneity of oocyte ooplasm, meiotic spindle location, size and shape of polar body, thickness of zona pellucida) [1]. However, oocytes with the similar morphology can have different potentials for further development. Others are cellular predictors and biomarkers of follicles (apoptosis activity in granulosa and cumulus cells, ratio of estradiol/testosterone, levels of IGF-1, IGFBP-1, leptin, inhibin, activin a, anti-Müllerian hormone in follicular fluid) and molecular markers of oocytes and cumulus cells (G6PDH and phosphodiesterase 3 activity, intracellular level of glutathione, cumulus gene expression profiling) [1]. The task of searching for new markers of oocyte maturity and quality is of great applied interest for developmental biology and reproductology.

One of conservative functions of serotonin (5-hydroxytryptamine, 5-HT) is regulation of oocyte maturation and spawning. It was shown that serotonin can promote oocyte maturation in mollusks [2,3,4], crustations [5], nemertean [6], echinoderms [7], fish [8,9], amphibians [7,10], and mammalians [10,11,12].

The molecular mechanisms of activation of oocyte maturation by serotonin vary greatly in different species. In most cases described, the action of serotonin is mediated by the activation of one of the specific serotonin receptors on the surface membrane of the oocyte [13]. Thus, in frogs and mice, serotonin causes the arrest of meiosis through its action on the 5-HT_7_ receptor coupled to the G_s_-protein, which stimulates the adenylate cyclase system of second messengers [10]. In bivalve mollusks, serotonin, by activating the membrane receptor on the surface of the oocyte, causes an increase in the cytoplasmic Ca^2+^ level and triggers the maturation of the oocyte [14]. The expression and activity of serotonin receptors and other components of the serotonergic system have also been described in maturing mammalian oocytes [15,16]. In isolated mouse ovarian follicle cultures, serotonin increases the expression of cyclin D1 and follicular cell maturity markers, as well as the oocyte growth factor GDF9 [17]. However, most of the effects of serotonin are abolished by fluoxetine [17], indicating that the SERT transporter is involved in the regulation of mouse oocyte maturation.

In our previous work, we showed that mouse oocytes can accumulate serotonin via membrane serotonin transporter (SERT) activity [18]. We demonstrated that the level of 5-HT intake varied in oocytes in morphologically similar follicles during the first wave of folliculogenesis in the ovaries of 14-day-old mice (14 dpp, days post-partum). These oocytes fell into two groups, depending on SERT activity, and formed a bimodal distribution: those which accumulated 5-HT on a high level, and those in which accumulation was similar to control group [19]. We suggested that differences in uptake levels in oocytes can correlate with positive or negative indicators of follicular selection. In this work, we investigated how SERT activity correlates with follicular selection and oocyte maturity.

## 2. Results

### 2.1. The Level of Serotonin Uptake by Oocyte Correlates with Granulosa Proliferation and Follicle Size

**Figure 1:**
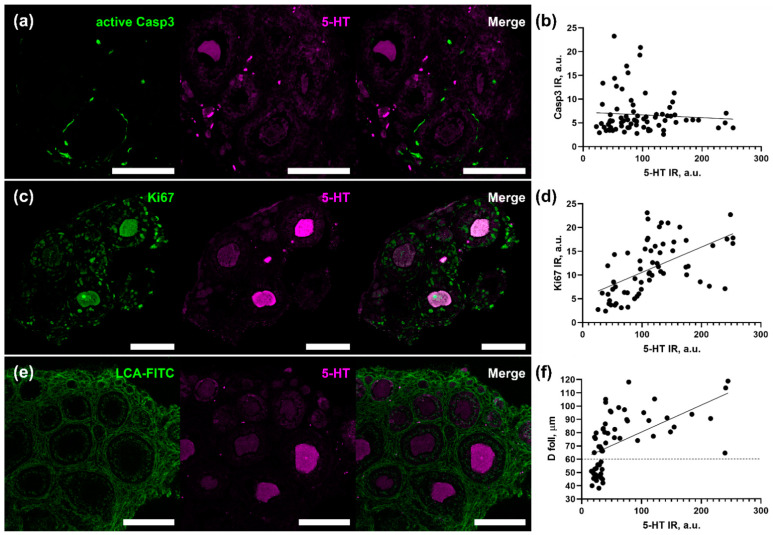
Correlation analysis of the intensity of serotonin accumulation by oocytes with morphological and functional indicators of the quality of ovarian follicles. (**a**,**c**,**e**) Cryosections of ovarian tissue of 14 dpp females incubated with 1 μM serotonin for 2 h, co-immunostained with antibodies against serotonin and an apoptosis marker—the active form of caspase 3 (**a**), a proliferation marker of Ki67 (**b**), and an LCA-FITC dye that reveals the boundaries of the follicles (**e**). Scale bar 100 µm. (**b**,**d**,**f**) Correlation matrices reflecting the interdependence of the degree of accumulation of serotonin by oocytes with the level of apoptosis (**b**) and proliferation (**d**) in follicular cells, as well as with the size of the follicle (**f**).

In order to find out if 5-HT uptake level in oocytes of growing follicles correlates with oocyte quality and developmental potential, we performed immunostaining of ovarian tissue after a 2 h incubation with 1 μM of serotonin. Cryosections of the ovarian fragments were immunostained by anti-5-HT antibodies to reveal serotonin uptake activity by oocytes, together with immunodetection of the level of apoptosis (Figure 1a) and proliferation (Figure 1c) in the layer of granulosa cells, as well as an assessment of the morphological parameters of follicles (Figure 1e).

The level of apoptosis was detected by antibodies against the active form of caspase 3 and, for each follicle, the level of active caspase 3 immunoreactivity in the layer of granulosa cells and the level of serotonin accumulation in oocytes were measured. Correlation analysis of the data obtained did not reveal a relationship between the activity of serotonin uptake and the level of apoptosis in the follicle (Spearman’s rank r = 0.13, *p* = 0.24) (Figure 1b).

Similarly, the level of Ki67 immunoreactivity in the layer of granulosa cells and the level of serotonin accumulation were measured in each particular follicle. Correlation analysis of the data obtained (Figure 1d) revealed a positive correlation between the activity of serotonin uptake by oocytes and the degree of proliferation of surrounding granulosa cells (Spearman’s rank r = 0.64, *p* < 0.0001) (Figure 1b).

Correlation analysis of serotonin uptake activity with morphometric parameters of follicles was performed on cryosections stained with LCA-FITC dye (Figure 1e). Both the level of 5-HT immunoreactivity in oocytes and the diameter of the same oocytes and follicles were measured with the subsequent correlation analysis. Spearman’s rank analysis revealed positive correlations of 5-HT uptake by oocytes with both oocyte diameter (r = 0.61, *p* < 0.001) and follicle diameter (r = 0.73, *p* < 0.0001) (Figure 1f). In the case of follicular diameter, it was shown that small follicles (diameter less than 60 µm) accumulate serotonin on a low level, while larger follicles vary greatly in 5-HT uptake ability. That indicates that serotonin transporter activity increases with the follicular growth.

These results indicate that the difference in the 5-HT intake activity in oocytes does not correlate with markers of atretic processes but correlates with positive markers of follicular growth, such as granulosa proliferation and follicle size.

### 2.2. SERT Activity Increases as the Oocyte Matures

**Figure 2:**
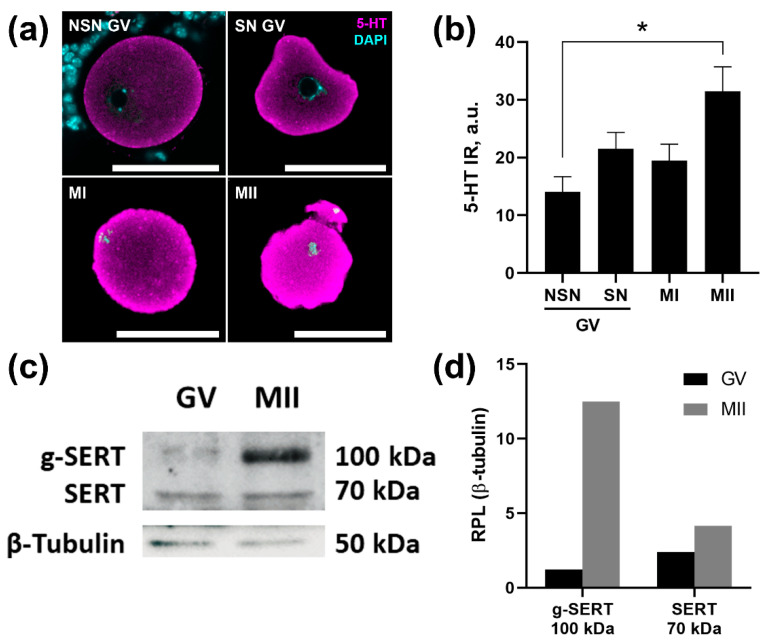
Serotonin uptake activity in mouse oocytes of different maturation stages. (**a**) Immunohistochemical detection of serotonin in oocytes at different stages of maturation after 2 h of incubation with 1 µM serotonin. Nuclear staining with DAPI was used to reveal the structure of chromatin in GV-oocytes: non-surrounded nucleolus (NSN) and surrounded nucleolus (SN). Scale bar 50 µm. (**b**) Quantification of anti-serotonin immunoreactivity in oocytes at different stages of maturation after 2 h of incubation with 1 µM serotonin. Asterisk indicates significant differences between groups using Kruskal–Wallis test with Dunn’s multiple comparisons test, * *p* < 0.05. (**c**) Western blot analysis of the content of immature (70 kDa) and glycosylated mature (100 kDa) forms of SERT in GV- and MII-oocytes. (**d**) Western blot data quantification. RPL—relative protein level normalized to β-tubulin protein content.

To test the relationship of SERT activity with the degree of oocyte maturity, the level of 5-HT uptake by oocytes at different stages of maturation was assessed after a 2 h incubation with 1 μM of serotonin and its subsequent immunodetection (Figure 2a). Low competence GV-oocytes with NSN-conformation of chromatin accumulate serotonin the least actively. SN GV-oocytes, which are considered more mature and promising, accumulate serotonin 1.6 times more actively. Approximately the same level of accumulated serotonin is found in a small number of MI-oocytes that have undergone GVBD during isolation from the ovary. The most active uptake of serotonin occurs by MII oocytes isolated from the oviducts. The average level of immunoreactivity observed in mature MII oocytes is approximately 2.3 times higher than that in NSN GV-oocytes.

### 2.3. The Mature Form of the SERT Protein Is More Expressed in Mature Oocytes

To find out if increased 5-HT uptake activity in MII-oocytes appears due to an elevated expression of the serotonin transporter, we performed quantitative analyses of the SERT protein content in GV- and MII-oocytes collected after superovulation protocol. Western blot analysis showed that the expression of glycosylated SERT protein in mature MII-oocytes is about 8 times higher than in GV-oocytes (Figure 2b). These results suggest that expression and activity of SERT in oocytes increases with oocyte maturation.

### 2.4. Fluoxetine Reduces Serotonin Content in Maturing Oocytes

**Figure 3:**
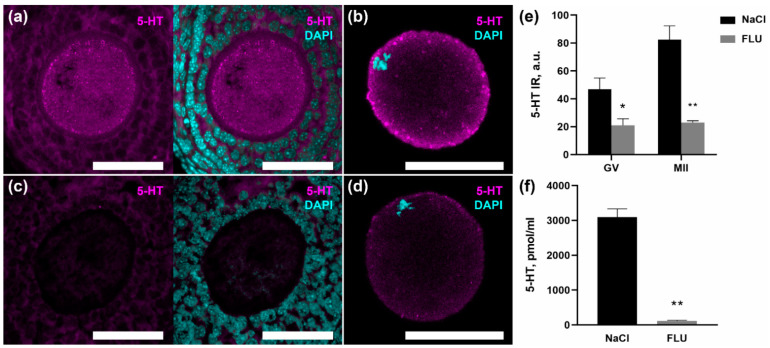
Effect of 7-day administration of fluoxetine (20 mg/kg) on serotonin content in oocytes. (**a**–**d**) Immunohistochemical detection of serotonin on ovarian tissue cryosections (**a**,**c**) and in ovulated MII-oocytes (**b**,**d**) of control (NaCl) (**a**,**b**) and experimental (FLU) (**c**,**d**) females. Scale bar 50 µm. (**e**) Quantification of anti-serotonin immunoreactivity in GV- and MII-oocytes of control (Ctrl) and experimental (FLU) females. Asterisks indicate significant differences between NaCl and FLU groups using Mann–Whitney test, * *p* < 0.01, ** *p* < 0.0001. (**f**) HPLC analysis of the serotonin content in the blood serum of control (NaCl) and experimental (FLU) females. Asterisks indicate significant differences between groups using Mann–Whitney test, ** *p* < 0.005.

In order to assess the possible developmental implications of disruption of the described mechanism, we tested the effect of the selective SERT inhibitor fluoxetine on the serotonin content in mature and immature oocytes. After a 7-day administration of fluoxetine, the content of serotonin in the blood serum of females is significantly reduced, amounting to only 4% of the norm (Figure 3e). The immunostaining of serotonin in the ovarian tissue showed that the content of serotonin in the oocytes of growing follicles decreases (Figure 3a,c). Also, a significant decrease in immunoreactivity is observed in mature MII-oocytes isolated from the oviducts of mice receiving fluoxetine (Figure 3b,d). It is important to note that against the background of a decrease in the total content of serotonin, immunopositive granules localized in the cortical region of oocytes also disappear. The data obtained clearly indicate that the mechanism of specific membrane transport of serotonin normally ensures the accumulation of serotonin in maturing oocytes and can be considered as a promising positive marker of their mature status.

## 3. Discussion

The study of characteristics that may be associated with a different level of activity of the accumulation mechanism is of interest in connection with the search for new factors responsible for determining the dominant follicles, oocyte maturation, and future development. In our previous work, we developed a model of 5-HT accumulation in the growing follicles of 14-day-old immature mice [18]. Followed by the first wave of follicular growth, there appear multiple preantral follicles in the ovary at that stage. We noticed that oocytes from morphologically similar preantral follicles had differences in activity of membrane transport of serotonin and formed a bimodal distribution [20]. In this study, we showed that oocytes obtained from 14-day-old mouse ovaries also have differences in serotonin uptake (Figure 2a), which indicates that this largely depends on the activity of the SERT in the oocytes themselves, and not on surrounding granulosa cells and other ovarian tissue.

The process of follicular selection in the ovary affects all stages of folliculogenesis [21]. The precise mechanism of how dominant follicles are being chosen has not been revealed yet. The list of factors supporting follicular growth or promoting atresia continues to grow. Together, they make up a complex system, the components of which regulate each other in a fine way. The transition to the antral follicle, the process in which gonadotropin sensitivity occurs, is the stage most vulnerable to the atresia [22]. Since 14 dpp is the age in which first antral cavities appear in the growing follicles [23], the revealed difference in the ability to accumulate serotonin observed here can be involved in the processes of the follicular selection. A number of features characterize the functional state of the preantral follicle. Important factors that were evaluated in this work were the morphological parameters of follicles, as well as the level of proliferation and apoptosis in the granulosa cells of preantral follicles. We revealed correlations between the level of SERT activity in oocytes and positive markers of follicular selection, namely follicle size and proliferation level in granulosa cells. It is known that oocyte quality and developmental potential depend on the size of the follicle, which is shown by in vitro studies in mice [24], but it should be taken into consideration that, taken alone, follicular morphology should not be used as a marker of good oocyte quality, as follicles of the same diameter can have different statuses [1]. Oocytes in preantral follicles are still growing [23], so the size of oocyte can also reflect differences in maturation stage. Oocyte regulates granulosa cell proliferation by secreting factors (GDF-9 and BMP-15 are playing key role) [25], which means that proliferation level in those cells depicts oocyte and follicular status. Taken together, these results support the idea that oocytes with high SERT activity are supposed to have better quality and developmental potential.

In this study, we showed that matured MII-oocytes have the highest degree of SERT activity (Figure 2a). Moreover, the trend of positive association of serotonin uptake activity with oocyte quality indicators is also true for two types of GV-oocytes: SN-oocytes had higher accumulation level than NSN-oocytes. SN-oocytes are considered to be more mature and perspective [26]. As 14 dpp is pre-puberty in mice, all oocytes in the ovary are NSN-oocytes, while SN-oocytes are observed in larger antral follicles [27]. We showed that, at early stages of follicular growth, GV-oocytes had low level of 5-HT accumulation (Figure 1f). This may be due to the activity of the functional barrier for serotonin in granulosa cells, which regulates the level of serotonin in the follicle, and apparently limits the threshold of serotonin uptake by the oocyte [28].

It is known that the concentration of serotonin in oviducts is higher than in the ovary in rats [29]. During ovulation, meiosis resumes in oocytes in the process of preparation for fertilization. The fact that SERT is more active in the MII-oocytes obtained from oviducts after ovulation than in those in the ovary suggests that its activity may be necessary for the later stages of oocyte maturation, fertilization and future development. We assume that the period after ovulation and before implantation could be the window of maternal influence on development through serotonergic mechanisms. Serotonin signaling specific membrane 5-HT receptors activity is involved [30]. Recent studies show that serotonin, after being captured inside the cell, can also provide intracellular regulation via the process called serotonylation, in which 5-HT molecules bind to certain proteins, irreversibly changing their function [31]. One of the suggestions about the purpose of serotonin accumulation in oocytes via SERT is that 5-HT can serotonylate some oocyte proteins, representing direct maternal impact on the offspring, but this hypothesis remains to be tested.

The importance of this mechanism is due to the fact that SERT is a target molecule for treatment of variable mood disorders by drugs called selective serotonin reuptake inhibitors (SSRIs) that are considered to be the most effective and safe antidepressants [32]. In this study, we used one of the most prescribed SSRIs throughout the world, fluoxetine [33]. Despite SSRIs’ ability for rapid inhibition of SERT activity [34], efficacy of treatment can be seen only after chronic administration, due to neuroadaptive changes. Serotonin levels in platelets decrease after prolongated treatment with fluoxetine [35]. Earlier, we demonstrated that the main source of serotonin in the ovarian follicle is maternal 5-HT from the bloodstream, as no synthesis appears in any cell type of the follicle [18]. Thus, there are no mechanisms in the ovary by which it would be possible to compensate for the action of SSRIs. In this work, we showed that, after 7 days of fluoxetine administration (20 mg/kg), serotonin levels in serum decreases up to 96%, with a following decrease in the serotonin level both in GV-oocytes in the ovary and MII-oocytes obtained from the oviducts. We also saw the disappearance of serotonin-immunopositive granules that are present in the cortical layer of cytoplasm of the oocytes in the control (Figure 3). The fact that 5-HT in the oocytes is located inside of granules can indicate serotonin signaling activity, provided presumably via VMAT2, of which expression was found in oocytes [16]. We assume that after chronic fluoxetine administration there is a lack of serotonin in the bloodstream that affects 5-HT levels, both in ovaries and oviducts. As we also showed that MII-oocytes had high level of expression of the mature glycosylated form of SERT protein, we suggest that fluoxetine could also inhibit SERT on oocyte’s membrane, which can also have impact on 5-HT concentration inside these cells. Considering the important role of maternal serotonin for normal development [36], we assume that this dramatic decrease of 5-HT in blood and periphery tissues can have a negative outcome on early development.

It should be noted that the dosage of fluoxetine used in this work (20 mg/kg/day) is rather high compared to those used in human therapy. However, it is this concentration of the drug that is effective in mouse models, inducing behavioral effects and maintaining drug levels comparable to those observed in humans with long-term use of SSRIs [37]. Due to the fact that the work was performed on a mouse model, before extrapolating them to humans, the results should be tested on human oocytes. However, we are convinced that our results indicate that puberty, following oocyte maturation, processes of ovulation, fertilization, and early development are the vulnerable periods for SSRI treatment in women.

## 4. Materials and Methods

### 4.1. Experimental Animals and Chemicals

Female C57BL/6 mice from the Laboratory Animal Center of the Koltzov Institute of Developmental Biology RAS, were used for the animal experiments. Animals were kept under controlled conditions (22–24 °C and 14L:10D photoperiod). Mice were given ad libitum access to food and water. The chemical used in the study was 5-HT-creatinine sulfate (H7752 Merck KGaA, Darmstadt, Germany). Experiments were performed in accordance with the Council of the European Communities Directive of 24 November 1986 (86/609/EEC). All protocols of animal experiments were approved by the Commission on Bioethics of the Koltzov Institute of Developmental Biology of the Russian Academy of Sciences.

### 4.2. Superovulation Protocol and Oocyte Collection

For the protocol of hormonal stimulation, mature mice were taken at the age of 2 months. In the interval of 3–4 PM a subcutaneous injection of PMSG (5 IU/mouse) was made. A total of 40–46 h after the injection of PMSG, an injection of hCG (5 IU/mouse) was made at 1–2 PM. The ovaries and oviducts were isolated 20 h after hCG injection, and washed from blood in dPBS cooled to 4 °C. Ovaries and oviducts were placed in L-15 medium (Thermo Fisher Scientific Inc., Waltham, MA, USA) preheated to 37 °C. GV-oocytes were released by rupturing antral follicles in the ovaries with a 29G needle. MII-oocytes were isolated by dissection of the ampullary parts of the oviducts. The obtained cumulus enclosed oocytes (GV) and oocyte-cumulus complexes (MII) were collected using a Stripper pipette and transferred to the four-well plate (Thermo Fisher Scientific Inc., Waltham, MA, USA) with L-15 medium supplemented with hyaluronidase (80 UI/mL) for 15 min. Then, the denuded oocytes were washed three times for 5 min in L-15 medium.

### 4.3. Ovary Fragment and Oocyte Incubation Experiments

In the case of tissue fragments experiments, ovaries of 2-week-old (14 dpp) mice were placed on ice in Dulbecco’s PBS buffer (Biosera, Nuaille, France) and washed to remove blood. The ovaries were placed in pre-warmed Hank’s saline buffer (Biosera, Nuaille, France) and dissected into eight pieces each. The ovarian fragments or isolated oocytes were incubated for 2 h at 37 °C in four-well culture dishes containing 1 mL of L-15 medium (Thermo Fisher Scientific Inc., Waltham, MA, USA), supplemented with serotonin (1 μM). The ovarian fragments or oocytes were fixed in 4% paraformaldehyde at 4 °C overnight.

### 4.4. Immunohistochemistry

The 5-HT immunostaining of ovarian cryosections and isolated oocytes was performed as described [18]. Samples were fixed with 4% PFA, and isolated oocytes were then treated with 0.5% SDS for 1 min to remove the zona pellucida to facilitate the unhindered penetration of antibodies into oocytes. Then, samples were permeabilized by PBS (PanEco, Moscow, Russia) with 0.1% Triton X- 100 (Merck KGaA, Darmstadt, Germany) (PBST), then blocked with 2% normal goat serum (Merck KGaA, Darmstadt, Germany), 1% BSA (Merck KGaA, Darmstadt, Germany), and 0.1% cold fish skin gelatin (Merck KGaA, Darmstadt, Germany) in PBST, and stained (overnight, 4 °C) with rabbit anti-5-HT antibodies (1:1000 S5545 Merck KGaA, Darmstadt, Germany), rat anti-Ki67 antibodies (1:1000 14-5698-82 Thermo Fisher Scientific Inc., Waltham, MA, USA) or rabbit anti-caspase 3 (active) antibodies (1:500 C8487 Merck KGaA, Darmstadt, Germany). After washing in PBST, samples stained with goat anti-rabbit IgG antibodies conjugated with CF 568 (1:500 sab4600085 Merck KGaA, Darmstadt, Germany), and donkey anti-rat IgG antibodies conjugated with CF 633 (1:500 sab4600133 Merck KGaA, Darmstadt, Germany). For ovarian morphology, sections were stained with Lens culinaris agglutinin (LCA) conjugated with FITC (1:1000 L32475 Thermo Fisher Scientific Inc., Waltham, MA, USA); for detection of GV chromatin configuration, oocytes were stained with DAPI (Merck KGaA, Darmstadt, Germany). Samples were then washed with PBST 3 times, and mounted in Mowiol for subsequent confocal laser scanning microscope analysis. For the subsequent quantification of immunoreactivity, all comparison groups were conducted in parallel, and cryosections of all samples within one experiment were stained on the same glass. In the case of immunodetection of serotonin and caspase 3, consecutive cryosections containing the same follicles were placed on two glass slides to use two rabbit antibodies in parallel.

### 4.5. Image Analysis

We analyzed the samples using a confocal laser scanning microscope Zeiss LSM 880 Airyscan (Carl Zeiss, Jena, Germany). All samples in the experiments were analyzed on the same microscope and software characteristics: objectives, pinhole, laser intensity, and detector sensitivity values. Photomicrographs were analyzed using FIJI software ImageJ 2.9.0/1.54f (open- source project). Immunoreactivity levels were quantified based on fluorescence intensity using the mean gray level instrument for the regions of interest (oocytes or granulosa cells layer).

### 4.6. Western Blotting

MII- and GV-oocyte samples (50–100 oocytes) were lysed in RIPA buffer, and the protein concentration was measured by the BCA Pierce™ Protein Assay Kit (Thermo Fisher Scientific, Waltham, MA, USA). Samples were denatured in Laemmli buffer for 30 min at 37 °C. Proteins were separated by 10% SDS-PAGE and then electrophoretically transferred onto Immun-Blot^®^ PVDF Membrane (Bio-Rad Laboratories, Inc., Hercules, CA, USA). The membranes were blocked with 5% BSA for 1 h at room temperature, and incubated with primary goat anti-SERT (1:10,000 ab130130 Abcam, Cambridge, UK) or mouse anti-β-tubulin (1:10,000 AC021 ABClonal, Woburn, MA, USA) antibodies for 1 h at room temperature. After washing in TBST, the membranes were incubated for 1 h at room temperature with horseradish peroxidase-conjugated donkey anti-goat IgG (1:50,000 sab3700284 Merck KGaA, Darmstadt, Germany) or goat anti-mouse IgG (1:50,000 115-035-003 Jackson ImmunoResearch Labs, West Grove, PA, USA) antibodies. After washing in TBST, conjugated antibodies were visualized using enhanced chemiluminescence in 0.1 M Tris-HCl, pH 8.5, 12.5 mM luminol, 2 mM coumaric acid, and 0.09% H_2_O_2_. The intensity of protein bands was measured using MII- and GV-oocyte samples (50–100 oocytes) (Bio-Rad Laboratories, Inc., Hercules, CA, USA). Expression levels were normalized to β-tubulin protein.

### 4.7. High-Performance Liquid Chromatography

Blood serum samples of 20 μL were dissolved in 200 μL 0.1 N HClO4 with 25 pmol/mL 3,4-dihydroxybenzylamine (Sigma-Aldrich, St. Louis, MO, USA), followed by centrifugation at 2000× *g* for 20 min. In the prepared samples, serotonin concentrations were measured using high performance liquid chromatography with electrochemical detection (HPLC-ED), as previously described [38].

### 4.8. Statistical Analysis

The results were statistically processed with the GraphPad Prism 6.0 software package (GraphPad Software, San Diego, CA, USA). *p* < 0.05 was used as a significance criterion.

## Data Availability

Not applicable.
